# Elevated energy loss in diastolic left ventricular inflow corresponds to an increase in kinetic energy in patients with a repaired atrioventricular septal defect: Quantification from 4D Flow MRI

**DOI:** 10.1186/1532-429X-17-S1-O6

**Published:** 2015-02-03

**Authors:** Mohammed SM ElBaz, Emmeline Calkoen, Jos J Westenberg, Arno Roest, Rob J van der Geest

**Affiliations:** 1 Division of Image Processing, Radiology, Leiden University Medical Center (LUMC), Leiden, Netherlands; 2 Pediatric cardiology, Leiden university medical center, Leiden, Netherlands

## Background

Patients after atrioventricular septal defect (AVSD) repair may present abnormalities in valve morphology and subsequently develop altered left ventricular (LV) inflow patterns [1]. This may lead to energy loss and affect the kinetic energy in the LV. We aimed to quantify diastolic viscous energy loss and kinetic energy in the LV and to evaluate their association in AVSD-repaired patients compared to healthy controls using 4D Flow MRI.

## Methods

23 AVSD-repaired patients with NYHA class 1 and 2 (age: 20±8 years) and 23 healthy controls (age: 19±8 years) were included. All subjects (Table 1) underwent whole-heart 4D Flow MRI at 3T with free breathing, three-directional velocity-encoding of 150cm/s in all directions, spatial resolution 2.3×2.3×3.0-4.2mm^3^ and 30 retrospectively-gated phases reconstructed over one cardiac cycle. The LV cavity was manually segmented over diastolic phases from 4D Flow data. For each segmented phase, the kinetic energy within LV was computed as 1/2 mv^2^, with (m) as the mass representing the voxel volume multiplied by the density of blood (1.025 g/ml) and (v) as the 3-directional velocity from 4D Flow MRI. Total kinetic energy (KE) was then computed by integrating the computed kinetic energy over diastole. Using Navier-Stokes energy equations, non-turbulent viscous energy loss (EL) was evaluated in the LV as the integration of viscous energy dissipation over diastolic period as described previously [2] with blood assumed as an incompressible Newtonian fluid. Both KE and EL were normalized by the end-diastolic LV volume (EDV). A dimensionless ratio (EL/KE_avg_) was computed with KE_avg_ as the average kinetic energy over diastole. Measured parameters were compared using unpaired Wilcoxon rank sum test. Association between EL and KE was evaluated using Pearson's correlation.

**Table 1:** Quantitative parameters of viscous energy loss and Kinetic energy during diastole in AVSD-repaired patients compared to controls

	Control (N=23)	AVSD-repaired patients (N=23)
Age (yrs)	19 ± 8	20 ± 8

Heart rate (bpm)	87 ± 11	94 ± 15

EL (mJ/mL)	0.21 ± 0.07	0.29 ± 0.12^a^

KE (mJ/mL)	0.16 ± 0.07	0.24± 0.11^b^

(EL/KE_avg_) *	1.31 ± 0.43	1.22 ± 0.5^c^

^a^ p<0.05 , ^b^ p<0.01 and ^c^ p=0.3 * the dimensionless ratio (EL/KE_avg_) represents the ratio of the amount of kinetic energy lost through viscosity (EL) to the amount of kinetic energy(KE) which is still available (retained) during diastole e.g. (EL/KEavg)=1.3 means that the amount of lost kinetic energy due to viscosity is 1.3 times the amount of kinetic energy which is still retained from diastole. KE_avg_ represents the average KE available over diastole

## Results

**Figure 1:**
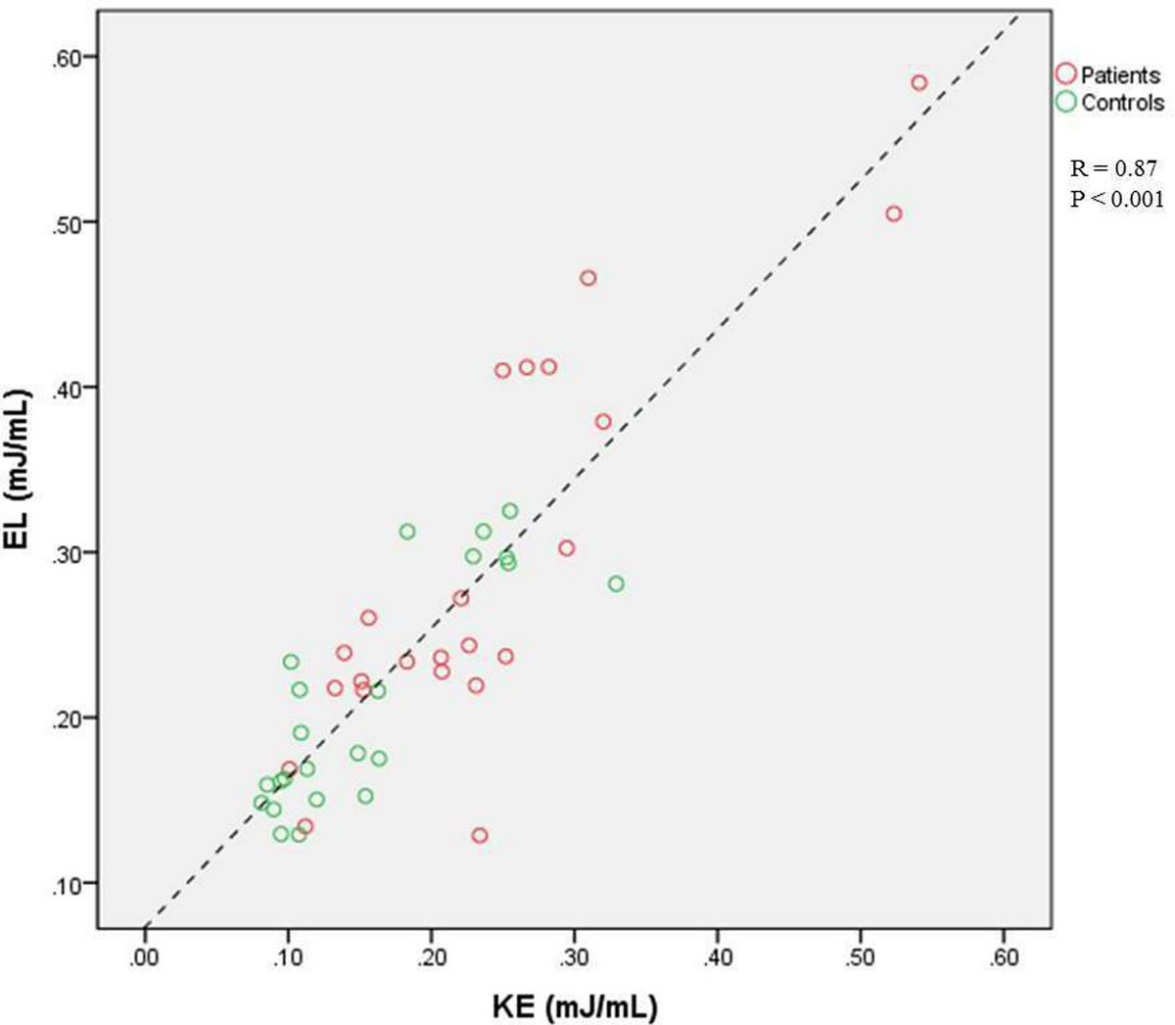
Correlation between kinetic energy (KE) and viscous energy loss (EL) in the AVSD-repaired patients marked in red and healthy controls marked in green. Both KE and EL are normalized by end diastolic volume (EDV). The dashed line correspond to the regression line.

Detailed results are presented in Table 1. During diastole, viscous energy loss (EL) was significantly higher in patients compared to controls (p<0.05). Additionally, a significant increase in KE was observed in patients compared to controls (p<0.01). However, the ratio between viscous energy loss and average KE over diastole was comparable in patients to controls (p=0.3). Strong correlation (R=0.87) was found between KE and EL (Figure 1).

## Conclusions

A significant increase in viscous energy loss (EL) is found in LV diastolic inflow of repaired-AVSD patients compared to healthy controls. This corresponded to higher levels of kinetic energy (KE) than controls. This parallel increase over diastole results in a ratio balance between EL and average KE comparable to controls. This could potentially be a mechanism of the heart to respond to the elevated levels of energy loss in the LV. This is one of the first *in vivo* studies to quantify and show the association between KE and EL from 4D Flow MRI. Further studies are needed to determine the impact of increased energy loss on cardiac function on the long term.

## Funding

MSM ElBaz and J.J.M. Westenberg are financially supported by a grant from the Dutch Technology Foundation (STW) project number 11626.

E.E. Calkoen is financially supported by a grant from the Willem-Alexander Kinder- en Jeugdfonds, M.S.
